# Revitalizing grand rounds in the time of COVID

**DOI:** 10.1186/s12909-024-06030-9

**Published:** 2024-10-03

**Authors:** Lekshmi Santhosh, Lee Anne Rosenstein, Robert M. Wachter

**Affiliations:** Present Address: Department of Medicine, University of California, 505 Parnassus Avenue, Box 0111, San Francisco, CA 94143 USA

## Abstract

COVID presented an opportunity to revolutionize the traditional format of Medical Grand Rounds (MGR). In this *Commentary*, we explore the educational ramifications of shifting MGR virtually with a focus on COVID-related content and its long-term sustainability. This transformation offers an inclusive interdisciplinary approach to sustain learner interest and improve education.

## Introduction

The pandemic transformed medical education, from the classrooms to the clinics, from undergraduate medical education (UME) to graduate medical education (GME) to continuing medical education (CME), representing opportunities to reimagine medical education [1]. While the transition to virtual learning was universal for UME, as directed by the Association of American Medical Colleges (AAMC) [2], the challenges posed by low levels of in-person attendance have bedeviled UME, GME, and CME educators long before the pandemic. A 2019 AAMC survey found that 28.8% of medical students “almost never” attended in-person lectures [3]. Resident and faculty noon conferences, including departmental conferences like Medicine Grand Rounds (MGR), had also been sparsely attended prior to 2020, victims of trainee work hours restrictions, work compression, and increased time outside the teaching hospital [4]. Moreover, residents were often on the frontlines of patient care during the pandemic, necessitating shifts in classroom and clinical learning and heightening the need to balance service with education.

During the early days of the COVID-19 pandemic, both the public and the profession struggled to find high-quality reliable sources of health information. Messaging from the CDC was often confusing and drowned out by misinformation online [5]. On March 19th, 2020, the UCSF Department of Medicine repurposed its MGR, which had been a traditional (and relatively sparsely attended) in-person affair covering a broad array of scientific topics, into a virtual-only “COVID Grand Rounds.” The goal was to present pertinent, timely, and accessible COVID-19 information to both professional and lay audiences in an online format that could even potentially increase educational engagement [6].

We hypothesized that transforming the traditional MGR format to a new virtual format and pivoting to COVID-related content could re-engage learners and faculty and combat broader public health misinformation. We also aimed to assess whether the educational impact of the changes forced by the pandemic would continue after the threat of the virus receded and people returned to a “normal.” The pandemic created a natural experiment to answer these questions.

## Methods

At baseline, Medical Grand Rounds were held in-person on Thursdays at noon, with typical audiences ranging from 25 to 50 people, with perhaps 100 attending a lecture by a prominent outside speaker. In March 2020, MGR shifted to a virtual-only format focused on COVID-19. As the pandemic receded, we resumed hybrid presentations and interspersed traditional MGR topics with COVID-focused content, settling into a once-a-month schedule for COVID grand rounds by mid-2021, with a further decrease to quarterly in 2022. COVID Grand Rounds featured invited speakers, many from outside UCSF, who spoke on topical COVID-related issues, with diverse representation from infectious disease specialists, research scientists, science communicators, policymakers, and epidemiologists. There were far fewer 50-minute lectures and more panel discussions, one-on-one interviews with newsmakers, reports from the field – formats that made sessions dynamic and interactive. Recorded videos were posted on YouTube within 48 h of the live event, allowing unrestricted viewing by the public and professionals alike.

We analyzed the number of virtual views of our institution’s virtual MGR from September 2021 to June 2023 and categorized topics into subspecialty categories. We analyzed metrics such as speaker gender and race, number of Zoom and YouTube views, self-reported gender and location of viewer, and view time. As MGR transitioned back to hybrid in-person and virtual formats, we recorded live in-person attendance, Zoom attendance, and YouTube views. No participants were contacted; only deidentified data (in-person attendance, Zoom attendance, and YouTube views were conducted.

## Results

**Fig. 1:**
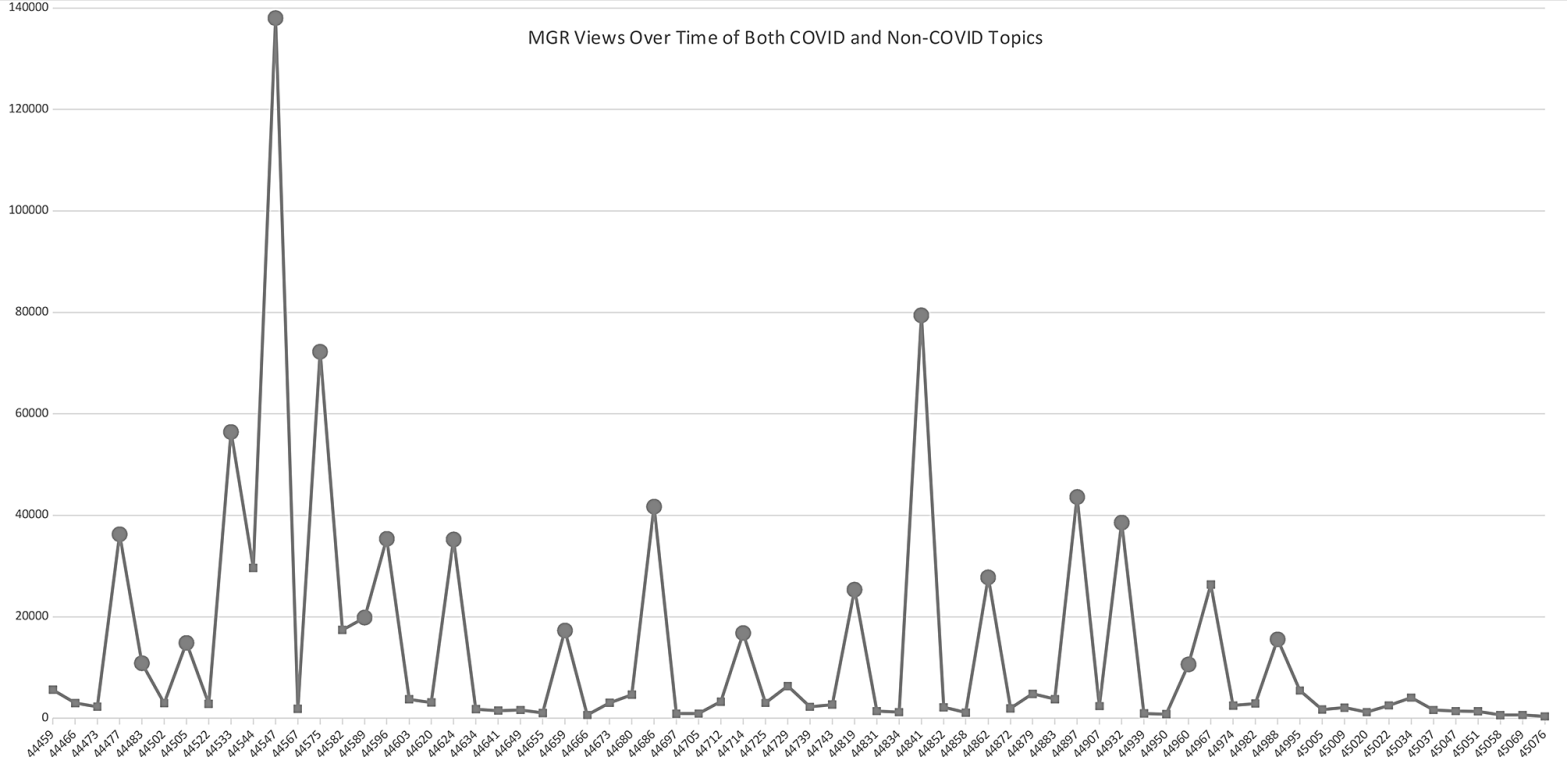
Panel **A**: Top ten UCSF medicine grand rounds topics, speakers, and views Panel **B**: MGR views over time of both COVID and non-COVID topics

The 10 most-viewed Grand Rounds on YouTube during the 2021–2023 timeframe all involved COVID-related topics, with the most-watched lecture (on the Omicron variant) being viewed over 138,000 times [Fig. 1A]. Of the “live” Zoom audience, 9 out of the 10 top viewed Grand Rounds were on COVID-related topics, with the 10th most popular lecture a February 2023 session on the science and implications of ChatGPT and artificial intelligence.

**Fig. 2:**
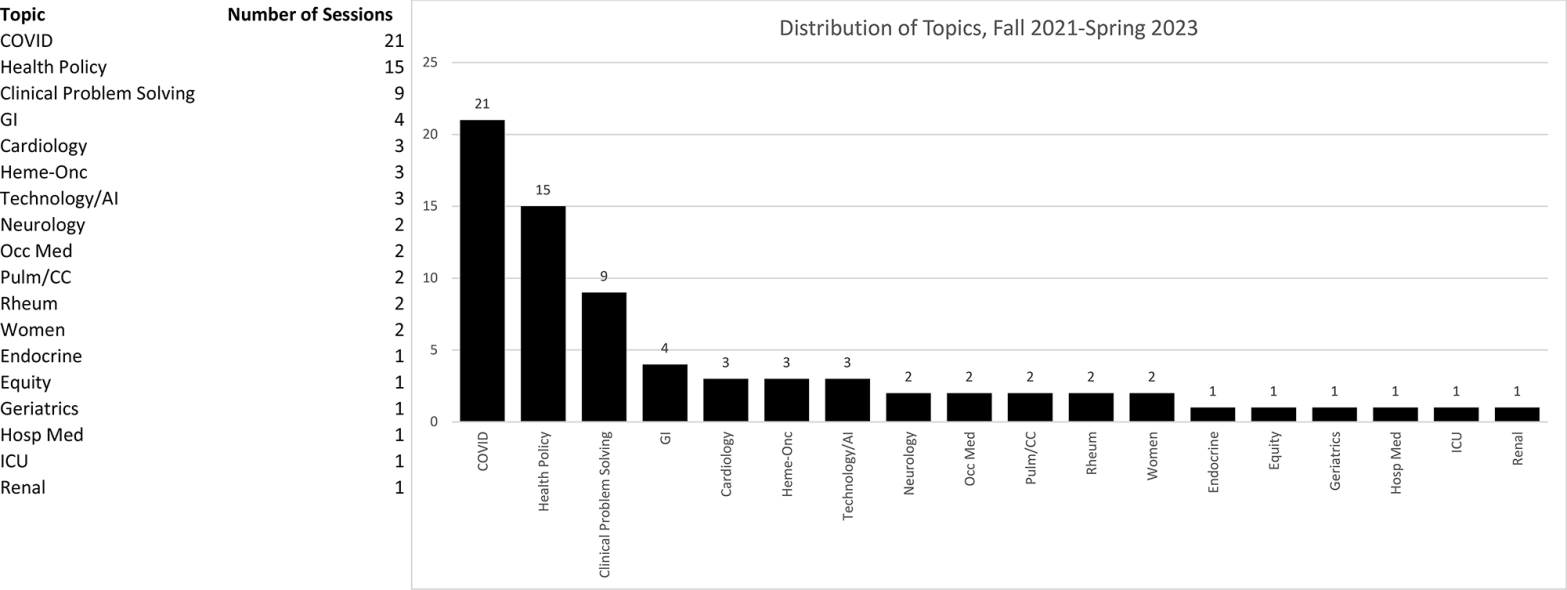
Distribution of topics, fall 2021 - spring 2023

Predictably, COVID was the most frequently covered topic (21/74), with other topics addressing the broad range of internal medicine specialties, as well as health policy, clinical reasoning, and women’s health [Fig. 2]. The majority (60.4%) of YouTube views of the top 10 most-viewed videos occurred within 10 days of posting the video online, and the largest number of viewers (22.2%) were in California, though videos were viewed by audiences around the world (33.4% international). Self-reported gender of viewers slightly skewed female (51.4%) and female viewers watched the videos for slightly longer than men [mean 14:43 vs. 12:25 min]. Viewers tended to be older than 45 years, and viewing duration increased steadily with viewer age [mean 16:26 min].

Longitudinal analysis of views [Fig. 1B] shows that viewership fell over time, but large spikes in viewership for COVID-related content continued; overall viewership remained substantially above pre-pandemic baseline levels. Unfortunately, robust data for pre-pandemic viewership are not available; in general attendance had been sparse with around 10–30 in-person attendees, depending on the topic. Future directions should explore the ongoing trends of in-person versus virtual attendance, the differences (if any) between engagement levels of in-person versus virtual attendees, and ideally, recall of medical knowledge or change in attitudes by in-person and virtual participants.

## Discussion

This natural experiment has important curricular lessons for post-pandemic didactic activities in general. Pivoting MGR to a virtual audience, shifting to a more engaging and interactive format, and focusing on timely and relevant COVID topics led to high levels of attendance and audience participation from both local viewers (many of them professionals and trainees at our institution) and a wider public and professional audience. Although COVID case numbers have declined worldwide, enthusiasm for COVID-related content remains high, and total attendance remains at least 2- to 3-fold higher than it was prior to the pandemic, even for non-COVID topics. Thus, COVID provided an opportunity to innovate and revitalize medical grand rounds.

Academic departments have long wrestled with the limitations of Grand Rounds [7] yet there have been few innovations in format and content. Steeped in tradition, most institutions’ grand rounds have not been subjected to rigorous assessment methods with an eye toward innovation to better meet learners’ needs and preferences. While traditional MGR formats tended to favor scientific advancements in one specific research area or subspecialty, COVID allowed the opportunity to provide education that was expansive and cross-cutting, synthesizing the fields of basic biology, pharmacology, history, sociology, epidemiology, political science, and more. We speculate that this broadened focus expanded the potential audience interested in MGR, and once viewers came for COVID content, they were more likely to stay for a lecture on the pathophysiology of the gut microbiome or the implications of ChatGPT, particularly since speakers were explicitly instructed to appeal to a broader audience and to employ more engaging formats.

From our natural experiment, we noted several best practices and lessons learned that may be generalizable and adaptable to other institutions. First, academic medical centers should consider explicitly sharing their expertise beyond the walls of their institutions to the general public, by using more accessible formats such as panel discussions, synthesizing cross-cutting topics, and adopting communication modalities that hybridize both in-person and virtual content. Speakers should be instructed to appeal to a broader audience and avoid overly specialized content. Speakers should be invited from a broad array of divisions and departments, bringing varying areas of expertise and attention to diversity, equity, and inclusion. When possible, social media should be used to amplify and disseminate learning points from speakers. Once momentum from increased attendance and engagement has begun, sustaining that momentum is critical with ongoing innovation and iterations based on feedback and reflection.

Study limitations include its single-site nature and the lack of baseline data assessing attendance learner satisfaction with the MGR series. YouTube views are an imperfect measure of engagement, as individuals may selectively watch parts of the recording rather than watching the entire hour in detail. Furthermore, we cannot ascertain whether most YouTube viewers were clinicians from other institutions versus members of the general public. Because COVID both led to the change in format and was the topic of a large fraction of conferences, disentangling how much of the increased viewership owed to format or topic is challenging. However, the fact that attendance remained significantly higher for non-COVID topics than it was during the baseline period reassures us that at least part of the improvement was due to format changes and is likely to be durable.

## Conclusions

Although the MGR audience has declined with waning interest in COVID, the new higher baseline interest suggests that learners are still engaged and interested in a broad array of topics, especially with a flexible hybrid format. Our natural experiment makes clear that new approaches to grand rounds can pay dividends. It also raises a key question: how can we create timely, relevant instruction that promotes continuing professional development across a variety of medical disciplines, while also being accessible to the public? More fundamentally, what is the purpose of grand rounds? As society inches closer to a “post-pandemic normal,” we believe that COVID prompted us to change MGR at our institution for the better. The challenge going forward will be to continue to innovate without the burning platform of a pandemic.

## Data Availability

The datasets used and/or analyzed during the current study available from the corresponding author on reasonable request.
